# Structure–Function Insights into the Fungal *Endo*-Chitinase Chit33 Depict its Mechanism on Chitinous Material

**DOI:** 10.3390/ijms23147599

**Published:** 2022-07-09

**Authors:** Elena Jiménez-Ortega, Peter Elias Kidibule, María Fernández-Lobato, Julia Sanz-Aparicio

**Affiliations:** 1 Department of Crystallography and Structural Biology, Institute of Physical-Chemistry Rocasolano, CSIC, 28006 Madrid, Spain; xelena@iqfr.csic.es; 2 Department of Molecular Biology, Centre of Molecular Biology Severo Ochoa, CSIC-UAM, 28049 Madrid, Spain; pkidibule@cbm.csic.es

**Keywords:** chitinase, crystal structure, chito-oligosaccharides, NAG6 binding mode, hydrolytic activity, specificity

## Abstract

Chitin is the most widespread amino renewable carbohydrate polymer in nature and the second most abundant polysaccharide. Therefore, chitin and chitinolytic enzymes are becoming more importance for biotechnological applications in food, health and agricultural fields, the design of effective enzymes being a paramount issue. We report the crystal structure of the plant-type *endo*-chitinase Chit33 from *Trichoderma harzianum* and its D165A/E167A-Chit33-(NAG)_4_ complex, which showed an extended catalytic cleft with six binding subsites lined with many polar interactions. The major trait of Chit33 is the location of the non-conserved Asp117 and Arg274 acting as a clamp, fixing the distorted conformation of the sugar at subsite –1 and the bent shape of the substrate, which occupies the full catalytic groove. Relevant residues were selected for mutagenesis experiments, the variants being biochemically characterized through their hydrolytic activity against colloidal chitin and other polymeric substrates with different molecular weights and deacetylation percentages. The mutant S118Y stands out, showing a superior performance in all the substrates tested, as well as detectable transglycosylation capacity, with this variant providing a promising platform for generation of novel Chit33 variants with adjusted performance by further design of rational mutants’. The putative role of Tyr in binding was extrapolated from molecular dynamics simulation.

## 1. Introduction

Chitin, the most extensive amino carbohydrate polymer in nature, is a linear polysaccharide made of repeated 2-acetamido-2-deoxy-D-glucose or N-acetyl-D-glucosamine (NAG) units, which occur as well-organized crystalline microfibrils. This polymer constitutes a fundamental component of fungi cell walls, exoskeletons of invertebrates and endoskeletons of molluscs [1]. Chito-oligosaccharides (COSs) are degradation products of chitin and chitosan (deacetylated form of chitin), soluble oligomers classified as fully acetylated (*fa*COS), partially acetylated (*pa*COS) or fully deacetylated (*fd*COS), depending on the chitinolytic material type from which they originated. Recently, COSs have attracted extensive biotechnological attention due to their high water solubility, temperature and pH stability, as well as their potential biological properties [2,3]. The biological activities of COSs have been related to their reactive amino/acetamido or hydroxyl groups, their degree of deacetylation (DD), degree of polymerization (DP) and pattern of acetylation (PA) [2,4]. In this sense, the enzymatic production of COSs is preferred over physical, chemical and electrochemical means, as it is a more controllable and less polluting production strategy. Currently, commercially available COSs are mostly obtained by hydrolysis of chitin and chitosan using concentrated acids [2,5].

Chitinases from the glycosyl hydrolases (GH) family 18 represent an ancient enzyme type found in nearly all kingdoms of life, even in organisms that are non-chitin producers (for an updated review, see [6] and references therein). Most fungal chitinases are included within this family, their general structure comprising an essential catalytic domain, a chitin-binding domain, a threonine-rich region and a C-terminal extension. However, not all are necessary for chitinase activity, with many enzymes encompassing only the catalytic domain [7,8]. In fungal GH18 chitinases, the DxxDxDxE and SxGG motifs correspond to the catalytic and substrate-binding site, respectively [9,10]. The signal peptide located at the N terminus is necessary for their secretion, as the chitinases lacking these sequences are cellular proteins participating in the microorganism morphogenesis process [11].

Structural studies of these enzymes have focused mostly on their catalytic domains exhibiting a general folding characteristic (β/α)8-TIM-barrel structure [12]. Besides this core, some GH18 enzymes contain an extra α + β insertion domain and are frequently named as bacterial-type chitinases, as they were first detected in these organisms, whereas those lacking this domain are named as plant-type chitinases. Both types present structural differences, especially at the substrate-binding cleft. In general, the substrate-binding site of fungal chitinases accommodates at least five sugar units, named −3, −2, −1, +1 and +2, with the cleavage occurring between the −1 and +1 positions [13]. Catalysis in GH18 chitinases exhibits some changes as compared to the typical double retaining mechanism, as instead of using a carboxylate side chain from the enzyme as the nucleophile, they use the acetamido group of the sugar at position −1 in a substrate-assisted mechanism [8,14], the activity thus depending on acetyl groups from the substrate. Moreover, along with their main hydrolytic activity, GH18 chitinases may exhibit a transglycosylation (TG) capacity, catalyzing the synthetic reaction that creates new glycosidic bonds between donor and acceptor saccharides.

Chitinases are further classified into *endo*- and *exo*-acting enzymes according to their cleavage type, with the first making random cuts in chitin chains, whereas the second degrade the polymer ends, generating primarily chitobiose (NAG2) [15]. In addition, many chitinases present processive ability, mostly producing disaccharides with relatively few odd-numbered saccharides, which is considered beneficial for the hydrolysis of crystalline chitin [16]. In general, *exo*-acting chitinases are processive enzymes that belong to the bacterial-type, whereas plant-type *endo*-acting chitinases are non-processive. The processive, *exo*-acting enzymes have been extensively characterized through many structural studies on bacterial enzymes that, combined with kinetic and molecular dynamics, provided key features of the catalytic machinery and the molecular determinants of processivity [6]. In contrast, the number of non-processive, plant-type *endo*-chitinases that have been structurally characterized is limited, with reported work on the fungal plant-type chitinases focused mostly on inhibitor design.

*Trichoderma* spp. are soil fungi commonly used in agriculture against plant pathogens due to their ability to produce different types of lytic enzymes, including chitinases. Among them, *T. harzianum* and *T. atroviride* are widely used as biocontrol agents [17]. Both species produce a mixture of *endo*- and *exo*-chitinases that act in a combined and processive way to efficiently degrade the crystalline nature of this polysaccharide. The *endo*-chitinase Chit33 from *T. harzianum* is included in the GH18 family. The gene *chit33* is comprised of 909 bp and code for a protein of 321 amino acids that was previously successfully expressed in *Pichia pastoris* [4]. This activity generated 1–7 units of NAG from the colloidal chitin hydrolysis, with the *fa*COS (GlcNAc)_4_ (also NAG4) being the main product [4]. This is interesting, as it was reported that a COS with more than four NAG units showed improved biological activity, although its synthesis was not well controlled [18].

To further expand its biosynthetic potential, we have solved the crystal structure of the *endo*-chitinase Chit33 and a complex of its inactivated D165A/E167A variant with NAG4. Depiction of its active site may provide the basis to engineer the molecular function through changes in protein stability, in the interaction with the biopolymers or change in the product specificity. Here, the structural analysis uncovers key residues for substrate binding and delivers suitable mutations to modify the Chit33 hydrolytic activity. The effect of proposed Chit33 mutations on several substrates with different polymerization and acetylation degrees was also evaluated.

## 2. Results and Discussion

### 2.1. Overall Folding of Chit33

**Figure 1:**
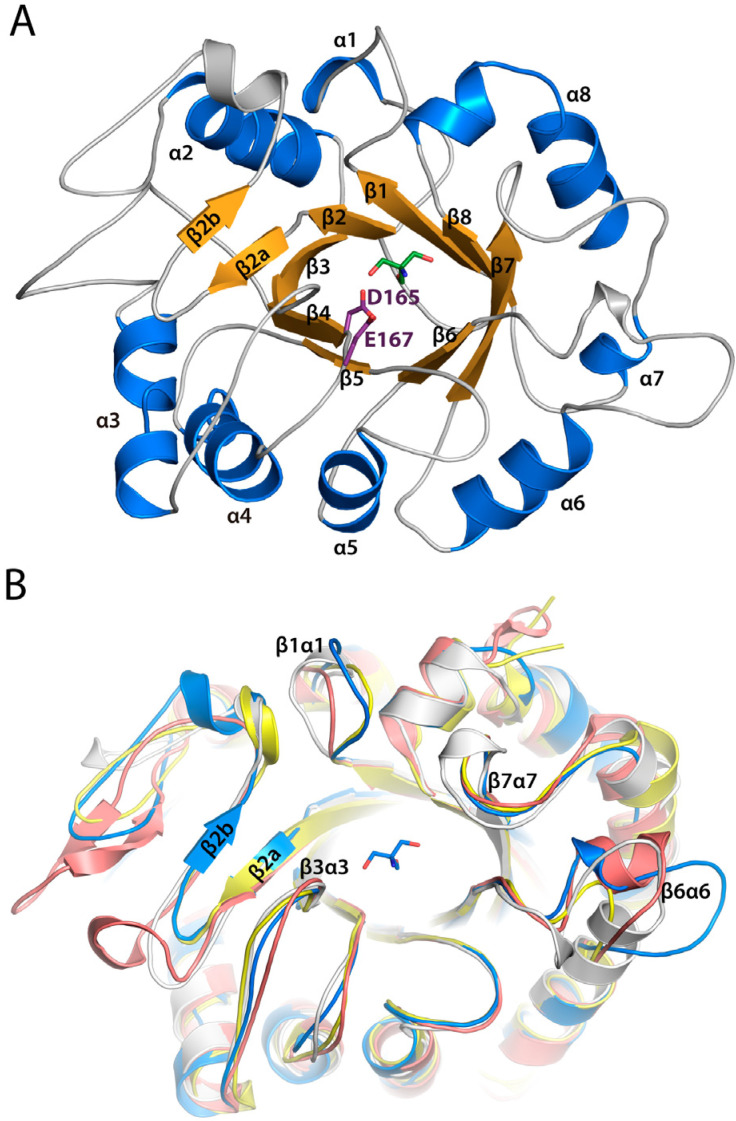
Chit33 folding. (**A**) Chit33 follows the (β/α)_8_ barrel topology, similarly to the GH18 family. Catalytic residues are colored as violet sticks. A Tris-buffer molecule, represented by green sticks, was captured in the catalytic center. (**B**) Structural superimposition of chitinase ScCTS1 in yellow (PDB code 2UY2), AfChiA1 from *Aspergillus fumigatus* in salmon (PDB code 2XVP) and hevamine from *Hevea brasiliensis* in gray (PDB code 2HVM) onto Chit33 wild type (blue).

To elucidate the molecular basis of its enzymatic activity, the crystal structure of native Chit33 was solved at 1.12 Å resolution (Figure 1A). Experimental details and structural determination procedures are presented in the Materials and Methods section and in Table S1. The crystals belong to the P 2_1_ 2_1_ 2_1_ space group, with one molecule in the asymmetric unit and 50% solvent content within the cell. The structure was determined by means of molecular replacement using the co-ordinates from the *Saccharomyces cerevisiae* chitinase 1, ScCTS1, as the search model [19]. The inactivated double-mutant D165A/E167A was also crystallized and used for soaking experiments with a series of chito-oligosaccharides (COS); however, only NAG4 was captured at the Chit33 active site, although a cocrystallization technique was also attempted with NAG3, NAG5 and NAG6.

Chit33 is folded into a (β/α)_8_ barrel domain, similarly to family 18 chitinases. A short and not well-defined α1, a bent α8, and the presence of two β-strand insertions after β2 are widespread features of other family 18 chitinases. Moreover, the short loops connecting the secondary structure elements are responsible for the definition of a tiny TIM barrel, which diverges from the bacterial-type chitinases embracing large βα loops, which even include a full chitin insertion α/β domain (CID). Furthermore, three disulfide bonds, Cys51-Cys103, Cys81-Cys93 and Cys200-Cys228, are detected in the Chit33 polypeptide chain.

The DxDxEx conserved catalytic motif is located at the end of strand β4 of the barrel, with Glu167 being the catalytic acid protonating the glycosidic bond and Asp165 playing a key role in assisting the acetamido group in its nucleophilic attack and stabilizing the developing positive charge on the oxazolinium ion. A TRIS molecule from the buffer solution was captured at the active site of the native crystals, making polar interactions through its hydroxyl groups with the catalytic Asp165 and Glu167, as well with Gln222 located at the end of β6.

**Figure 2:**
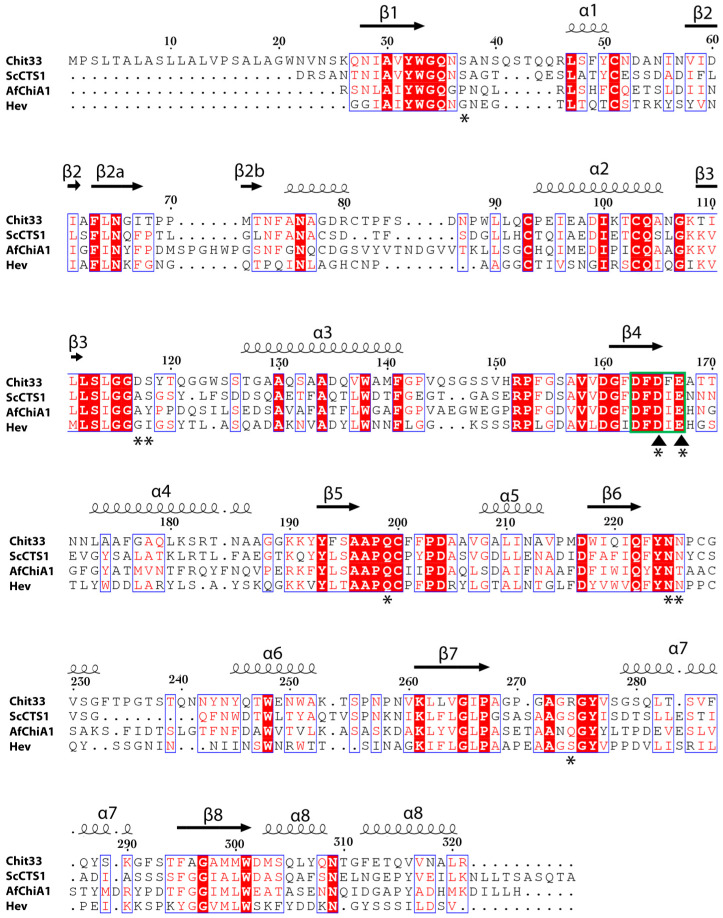
Sequence alignment of Chit33 with homologues. Sequences from the fungal chitinases from *S. cerevisiae* (ScCTS1) (PDB code 2UY2), *A. fumigatus* (AfChiA1) (PDB code 4TX6) and the plant chitinase hevamine from *Hevea brasiliensis* (PDB code 1HVQ) are shown (ESPript—http://espript.ibcp.fr, accessed on 8 October 2020). The DxDxE motif is boxed in green. Inverted triangles indicate catalytic residues, and asterisks mark residues mutated in this this work.

A comparative BLAST alignment of Chit33 sequence reveals closest homology to fungal chitinase ScCTS1 from *S. cerevisiae* (PDB code 2UY2, 45% identity) [19], AfChiA1 from *Aspergillus fumigatus* (PDB code 4TX6, 34% identity) [20] and the model plant chitinase hevamine from *Hevea brasiliensis* (PDB code 1HVQ, 32% identity) [21], all of them with a very conserved fold with RMSD between 1.4–1.9 Å after superimposition of their Cα atoms onto Chit33 (Figure 1B and Figure 2). All these proteins share rather short βα loops contouring an open, active site architecture, with major conformational differences found at loops β1α1 (Gly34-Arg46) and β6α6 (Phe223-Asn244), which are longer in Chit33, as well as the more structured β2α2 insertion of AfChiA1. In particular, the longer β1α1 loop makes a deeper cavity in Chit33, conferring a more tunnel-like appearance to its active site.

### 2.2. Substrate Binding Mode

**Figure 3:**
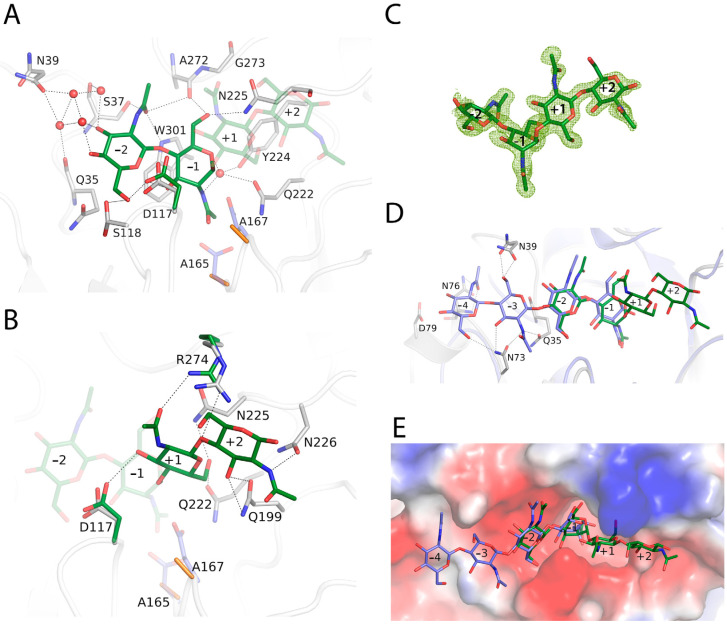
Structural details of the Chit33 active site. (**A**) Depiction of free and complexed Chit33 structures at the non-reducing (**A**) or reducing end (**B**). Catalytic resides are colored in slate/orange, and polar links are represented by dashed lines. Wild-type residues are represented by grey sticks, whereas residues showing conformational differences in the D165A/E167A-NAG4 complex are indicated by green sticks. (**C**) The final 2Fo-Fc electron density map at NAG4 moiety in the complex is contoured at 0.9 σ. (**D**,**E**) Extrapolation of additional binding subsites at the Chit33 non-reducing end by superimposing the hevamine-NAG4 coordinates (slate, PDB code 1KR0) onto the Chit33-NAG4 complex (grey and green). (**E**) Zoomed-in view of the molecular surface showing the electrostatic potential of the active site cavity.

To further investigate the substrate binding mode and to provide a platform for rational mutant design, we determined the crystal structure of the inactive double-mutant D165A/E167A with the substrate NAG4 (Figure 3). A clear electron density is observed for the tetrasaccharide, spanning subsites −2, −1, +1 and +2. All the subsites are highly defined though a large number of polar interactions that tightly fix the sugar.

At subsite −2, the CO from the N-acetyl moiety is strongly fixed by three direct hydrogen bonds to the Ala272 main-chain and to the side chains of Ser37 and Trp301. Moreover, a net of four water molecules connect O3 and O4 from NAG to Gln35 and Ser37 backbones and to the Asn39 side chain. Additionally, O6 from the sugar makes direct hydrogen links to Ser118 and Asp117 (Figure 3A).

At subsite −1, the boat sugar conformation postulated for the bound substrate in a state previous to oxazinium intermediate formation was crystallographically trapped. The stacking interaction with Trp301 is conserved in plant-type *endo*-chitinases, as it is critical for sugar deformation before the scission of the glyosidic bond (Figure 3). There is a water molecule that interacts with Gln222 and Tyr224 side chain and with the nitrogen atom from the N-acetyl glucosamine and putatively with the carboxylates from the catalytic Glu167 in the native protein, which could act as the reaction assistant to generate the oxazinium intermediate. In addition, O3 is hydrogen-linked to Asp117, both through its main and side chains, whereas O6 is polar-linked to the Ala272 backbone and to the Asn225 side chain.

Previous structural and kinetic studies on hevamine [22] proposed the combined action of Asp125 and Tyr183 (Asp165 and Tyr224 in Chit33) in positioning the carbonyl oxygen of the N-acetyl group at position −1 near to the C1 atom, which would stabilize the positively charged transient intermediate during substrate-assisted nucleophilic attack. Thus, Asp125 would be hydrogen-bonded to the nitrogen of the N-acetyl group, whereas its interaction with Tyr183 would fix the oxygen close to the target C1 atom. In our study, although we succeeded in trapping the distorted boat conformation of the substrate at subsite −1, these hydrogen-bonding interactions are lost in our double D165A/E167A mutant, which results in a flip of the N-acetyl group that locates its oxygen carbonyl away from the C1 atom. This reinforces the proposal that the Tyr is necessary but not sufficient to trigger the productive positioning of the N-acetyl moiety for efficient catalysis.

At the reducing end (Figure 3B), at subsite +1, the side chain of Arg274 interacts with the N-acetyl CO group and with the O4 glycoside bond, whereas Asp117 connects to the O3 atom through a hydrogen bond. Moreover, Gln222 and Asn225 side chains stabilize the O6. With regard to subsite +2, the Asn226 side chain is linked to N2 from the NAG moiety, whereas O3 is stabilized by a bifurcated hydrogen to the Gln199 side-chain.

Structural comparison of the wild type and the complex with NAG4 Chit33 does not show significant differences, apart from a visible reorganization of the Asp117 and Arg274 side chains that approach the substrate, enabling direct polar links. This observation may assign an important role in binding to these two residues that are not conserved in its homologues. Thus, Asp117 makes direct polar links to the sugar at subsites −2 and +1, possibly contributing to maintenance of the visibly bent arrangement of the bound substrate and the required productive boat conformation of NAG at subsite −1. Moreover, Arg274 links the N-acetyl group of sugar at subsite +1, as well as the glycoside-bond oxygen, suggesting a role in positioning the aglycone moiety of the substrate.

On the other hand, superimposition of hevamine complexed with NAG5 bound at the negative end of its active site [22] onto the Chit33-NAG4 structure allows for extrapolation of additional putative binding sites at the non-reducing end of the Chit33 active site (Figure 3D,E). These subsites would be defined by loops β1α1 and β2α2 of the barrel, with Gln35 and Asn39 from the first loop and Asn73 and Asn76 from the second, making many polar interactions with the NAG moieties located at putative subsites −3 and −4.

### 2.3. Structural Traits of the Chi33 Active Site

Chit33 belongs to class III (plant-type) enzymes characterized by the presence of shallow catalytic tunnels and open substrate-binding grooves. In contrast to the widely studied processive *exo*-chitinases mostly belonging to bacterial subfamily *A*, *endo*-chitinases reported to be active on crystallin chitin are still limited, needing to be further explored. Unlike the processive mechanism involved in chitin hydrolysis, in which aromatic residues are essential, structural analyses have revealed that this group of enzymes harbors fewer aromatic residues than the *exo*-chitinase counterpart. Thus, Trp301 at the base of the Chit33 –1 subsite is the sole tryptophan platform completely conserved in all family GH18 chitinases, including the broadly studied *exo*-chitinases SmChiA and SmChiB [23]. Moreover, as mentioned above, a tyrosine (Tyr224 in Chit33) was proven to be essential for deformation of the −1 ring in this family of chitinases [22]. However, Chit33 shares other characteristics with *exo*-chitinase catalytic clefts, such as the presence of a relatively extended catalytic tunnel defining additional recognition binding subsites at the non-reducing end, as seen in Figure 3D, E, as well as an apparently deeper cleft than its fungal homologues and hevamine.

Despite the high level of folding conservation within GH18 *endo*-chitinases, differences in the active site loops are mainly responsible for Chit33 specificity. In particular, β1/α1, β3/α3 and β7/α7 present a non-conserved amino acid composition that participates in substrate recognition, providing a higher number of polar interactions absent in the other homologous (Figure 2). Thus, a large number of lined polar interactions dominated by polar residues assists in substrate recognition, similarly to that previously described in the *exo*-chitinase Chit42 [24].

Regarding loop β1α1, the Ser37 side chain anchors the carboxyl moiety of the NAG at position −2, being exclusively conserved in the closest homologue, ScCTS1. Moreover, a longer Chit33 β1α1 loop enables the side chain of Asn39, unique to Chit33, to participate in substrate binding, probably making important links at putative subsites −3 and −4, as suggested. The double conformation observed in our crystals suggests an appropriate flexibility of these residues to fit the distal position of the substrate, an issue that is illustrated below.

On the other hand, the β3α3 loop does not apparently participate in any interaction at the catalytic tunnel of the Chit33 homologues, whereas Asp117, instead of the equivalent Ala/Gly, is involved in at least three hydrogen bonds with NAG units, spanning subsites −2, −1 and +1. Moreover, Ser118 is conserved in ScCTS1 but is substituted by Tyr125 in AfChiA1 or Ile82 in hevamine. Interestingly, the backbone arrangement of ScCTS1 prevents a polar link with the equivalent Ser, whereas the AfChiA1 Tyr125 side chain points away from the active site [20]. Consequently, Asp117 and Ser118 seem to be major features of Chit33 for substrate binding at the −2, −1 and +1 subsites, and both β1α1 and β3α3 loops appear to be responsible for the deeper cavity of the non-reducing end of Chit33 as compared to its homologues.

Remarkably, despite β7α7 having a rather conserved sequence, the presence of Arg274 in Chit33 promotes essential interactions at the reducing end (+1, +2) that might have been important to trap the substrate occupying the full catalytic cavity. Thus, the shorter Ser in ScCTS1 and hevamine or Asn281 in AfChiA1 partially maintains the polar character in this region, although unable to produce direct interactions with the substrate. Finally, loops β5α5 and β6α also define the catalytic groove, mostly through polar links with Gln199, Asn225 and Asn226 side chains, which are well conserved within Chit33 homologues.

In summary, the major structural trait of the Chit33 binding site is the presence of the non-conserved residues Asp117 and Arg274, which act as a clamp that may fix the bent conformation of the substrate that occupies the full catalytic groove, conversely to that reported in hevamine. Moreover, the unique Asn39 may provide additional putative binding subsites −3 and −4, which explains why the major product obtained from chitin hydrolysis was NAG4. Nevertheless, it is remarkable that we failed to trap the Chit33-NAG6 complex—an issue that can be explained by looking to the crystal packing, which shows that the non-reducing positions of the active site are blocked by the small loop following α8 (Ala187-Lys192) from a symmetry-related molecule. This blockage could not be eluded by cocrystallization of the complex instead of soaking preformed crystals into NAG6.

### 2.4. Mutational Changes Modulate Enzymatic Activity

**Figure 4:**
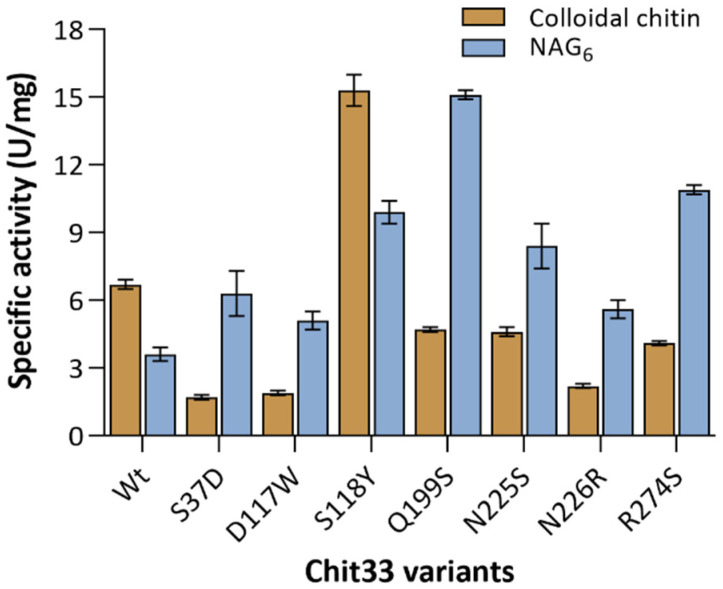
Hydrolytic activity on fully acetylated substrates of Chit33 variants including the indicated mutations. Specific activity data (U mg^−1^ of protein) are ab average of three independent experiments, and standard errors are indicated. Colloidal chitin (8 mg mL^−1^) and NAG6 (300 μM) were used as substrates.

The structural analysis of the Chit33-NAG4 complex was used as a starting point to select suitable mutations to modulate the enzymatic activity. Thus, changes in the sequence at β1α1 (S37D), β3α3 (S118Y, D117W), β5α5 (Q199S), β6α6 (N225S, N226R) and β7α7 (R274S) were introduced by site-directed mutations, and the resulting proteins were produced and biochemically characterized through its hydrolytic activity on colloidal chitin and the NAG6 substrate. As shown in Figure 4, all the mutants exhibit decreased activity on chitin, except the S118Y variant, which presents a twofold specific activity on the polymer. Additionally, contrary to observations in the wild type, all but S118 present a superior specific activity on the NAG6 chito-oligosaccharide than on the polymer. This observation can be explained by the fact that the non-processive chitinases need a more flexible catalytic machinery to compensate for the lack of processivity in degrading chitin. In this context, flexible polar residues at the active site may contribute to a dynamic on–off ligand binding, facilitating the entrance of the substrate and the product release after each catalytic cycle. Thus, the removal of these chains or disruption of their corresponding interactions in the tested mutants may have a negative influence specifically in the polymer, where the binding/releasing events may represent a limiting step. In turn, this possibly points to an additive contribution of all the polar links made with the ligand at the active site.

Therefore, the kinetic assays of the Chit33 variants (Table 1, Figure S1 in Supplementary Material) show that all the mutations maintained or reduced the catalytic efficiency (*k*_*cat*_/*K*_*m*_) on colloidal chitin, except the S118Y variant, with a threefold increase, mostly due to a higher affinity (*K*_*m*_) for this polymeric substrate. On the contrary, none of the changes apparently reduce the catalytic efficiency of the enzyme on NAG6, with the mutants S118Y Q199S, N255S and R274S showing a superior two- to fourfold catalytic efficiency of hydrolytic activity on NAG6 relative to the native enzyme, their decreased affinity being compensated with a superior catalytic constant (*k*_*cat*_).

Reactions were performed in triplicate, and standard errors are indicated. *K_cat_* was calculated from V_max_, considering 33 kDa for Chit33.

The hydrolytic activity of the mutants was also tested against different chitosans with varied molecular weight and DD (Table S2). As expected, the specific activity of the endo-chitinase Chit33 is higher on the chitosan with lower DD, which may be a direct consequence of the direct role of the NAG acetamido group in its catalytic mechanism. This trend is basically shared by most of the variants analyzed. Remarkably and as described above on chitin, the mutant S118Y stands out from the others, showing a five and ninefold increased activity compared to the wild type on CHIT50 and CHIT100, respectively. A final interesting observation is the S37D detectable activity on the CHIT600, an issue that might be further exploited to develop more variants capable of degrading this recalcitrant substrate.

**Figure 5:**
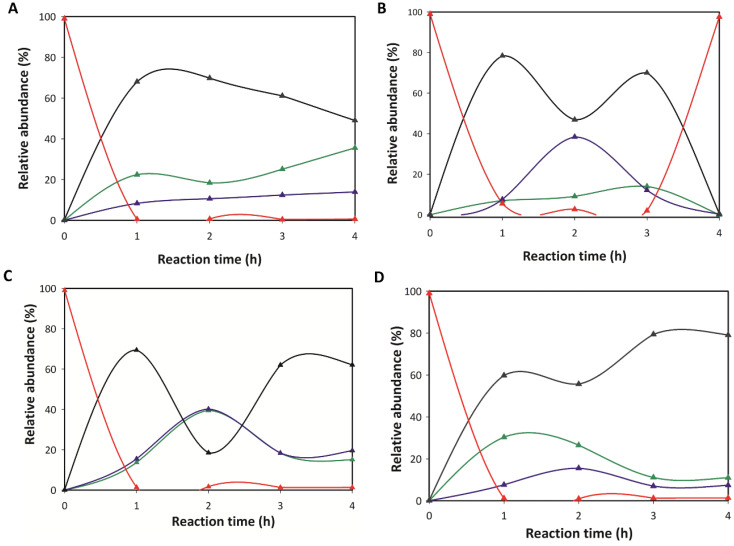
Time courses of NAG6 degradation catalyzed by wild-type Chit33 (**A**), S118Y (**B**), S37D (**C**) and D117W (**D**) variants. Relative abundance of the indicated NAG oligomer refers to total NAG derivatives detected by MALDI-MS in the reactions versus reaction time. Enzymatic reactions were conducted in 100 mM phosphate potassium, with a pH of 5.5 and 2.0 mM NAG6 at 45 °C. The color code is **NAG2**; **NAG3**; **NAG4**; **NAG6**. The results are represented as the average of two independent assays.

Finally, the effect of mutations on the potential transglycosylating activity of Chit33 was evaluated using NAG6 as substrate and monitoring the products formed during 4 h MS (Table S3 in Supplementary Materials). The final products after 4 h reactions are shown in Table 2, whereas Figure 5 illustrates the time course of COSs produced by four of the protein variants. With the sole exception of S118Y, the wild-type enzyme and all its variants hydrolyzed NAG6 mostly into NAG4, NAG2 and NAG3, with the first being the main product detected at all reaction times analyzed. NAG was not detected in any of the reactions, whereas traces of NAG5 were observed in all reactions.

Relative abundance of each NAG derivative refers to the total peak areas detected for all NAG oligomers in the reaction.

The almost exclusive presence of NAG6 after 4 h of reaction catalyzed by S118Y (Table 2) may be interpreted as an obstructed hydrolysis of this substrate in this variant, but inspection of Figure 5B reveals the production of NAG4, NAG3 and NAG2 along the time course of the reaction, with NAG4 and NAG3 showing a comparable relative abundance after 2 h. Thus, the subsequent replacement of NAG3 and NAG4 by NAG6 shown in Figure 5B is indicates transglycosylation, an activity that seems promoted in this variant at least after 4 h. However, only NAG4, NAG3 and NAG2 were detected after 24 h reaction catalyzed by all variants (Table S4 in Supplementary Materials), including S118Y, which means that the generated NAG6 is hydrolyzed later. Evidence of transglycosylating capacity is also detected in the time course of the reactions catalyzed by other variants, as shown for S37D and D117W in Figure 5C,D. Inspection of the corresponding product profiles indicates that the NAG4 initially produced from the hydrolysis of NAG6 is partially hydrolyzed after approximately 2 h, especially by the S37D variant, although the succeeding increment observed in NAG4 after 4 h must be generated by some transglycosylation activity. Consequently, the specificity of the transglycosylation activity of Chit33 may be modulated by mutation of relevant residues at the active site, an issue that might be further exploited for synthetic purposes.

### 2.5. Molecular Dynamics Evaluation of the Peculiar Hydrolytic Activity in the S118Y Mutant

According to our results, the mutant S118Y presents a superior hydrolytic performance against all the tested substrates and, apparently, a particular transglycosylating profile. As shown in the Chit33-NAG4 complex (Figure 3A), Ser118 presents a dual conformation in the free enzyme that is fixed upon ligand binding and orients to make a polar link to O6 from the sugar at subsite −2. Moreover, it is next to Asp117, which links to the sugars bound at subsites −1 and +2, both residues occurring at loop β3α3, which shapes the basis of the non-reducing end of the substrate. None of these residues are conserved, and as mentioned above, the Ser118 position is occupied by the bulky Tyr125 in the homologue AfChiA1. Previous structural studies [20] attributed conformational flexibility to this Tyr, which flipped out to accommodate large inhibitors. However, its role in binding was not well defined in the absence of substrate complexes. In this context, we selected Ser/Tyr substitution as a tempting choice to investigate its prominent potential effect on Chit33 activity and/or selectivity.

**Figure 6:**
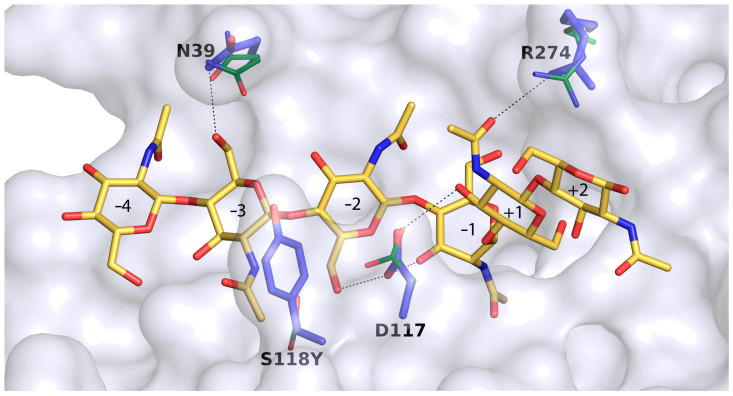
Molecular dynamics simulation of a putative Chit33-S118Y-NAG6 complex. Residues presenting conformational changes with respect to the Chit33-D165A/E167A-NAG4 complex are highlighted, showing the crystallographic position as green sticks.

In view of the positive results obtained through this change in the hydrolytic activity, we extrapolated the possible role of tyrosine in binding by means of a molecular dynamics simulation of a putative Chit33-S118Y-NAG6 complex modelled from the Chit33-D165A/E167A-NAG4 crystal structure, as explained in the Experimental section. The results are shown in Figure 6. First, it should be noted that subsites −4 and −3 present essentially the same features proposed and described above (Figure 3D,E). Additionally, most residues at the active site are in the same position as that observed in the crystallographic Chit33-NAG4 complex, with the exception of small conformational changes observed at Arg174 and Asp117, a residue that optimizes its interactions with sugar at subsites −2, −1 and +2, as well as Asn39, which presents a single conformation interacting with the sugar at subsite −3, as predicted.

The most interesting feature of this model is the location of the Tyr side chain very close to subsites −3 and −2 and allocating the N-acetyl group of the sugar at subsite −3 in a rather constrained pocket by stacking to its aromatic ring. Moreover, the Asp117-Tyr118 tandem outlines a narrow cavity that may fix the negative part of the substrate contributing to reaching the bent conformation required for hydrolysis to occur. Thus, a favorable positioning of the N-Acetyl group at subsite −3 and a closer interaction with the substrate at subsite −2/−1 may enhance substrate binding to the Chit33-S118Y active site cleft. In turn, the flexible Tyr side chain is not impeded from flipping out, possibly assisting in product release. This role of Tyr as a switch facilitating the entrance/release events may provide a rationale for the superior hydrolytic activity of this mutant as compared to the wild type. Additionally, an improved binding of the donor substrate at the negative end may stabilize the enzyme–glycosyl intermediate, which may also have a positive effect on the transglycosylating ability of the Chit33-S118Y mutant, as observed.

## 3. Materials and Methods

### 3.1. Materials

Chitin (from shrimp shells, coarse flakes; DD ≤ 8), N-acetylglucosamine (NAG) and chitosan CHIT50 (MW 50–190 kDa; DD 77%) were from Sigma Aldrich (St. Louis, MO, USA). Chitosan CHIT100 (MW 100–300 kDa; DD > 90%) and CHIT600 (MW600–800 kDa; DD >> 90%) were from Acros Organics (Thermo Fischer Scientific Inc., Waltham, MA, USA). N,N’,N’’,N’’’-acetyl chitotetraose (NAG4) and N,N’,N’’,N’’’,N’’’’,N’’’’’-acetyl chitohexaose (NAG6) were from Carbosynth Ltd. (Berkshire, UK). Colloidal chitin preparation was obtained as previously indicated [25]. Yeast nitrogen base w/o amino acids (YNB) was from Difco (BD, Sparks, MD, USA). All reagents were of the highest purity grade.

### 3.2. Strains, Expression Media, Plasmids and Site-Directed Mutagenesis

The yeast *Pichia pastoris* GS115 (*his4-*) from Invitrogen (Carlsbad, CA, USA) was used as expression host and was cultured at 30 °C and 250 rpm in YEPD medium (1% yeast extract, 1% peptone, 2% glucose; all *w*/*v*). The yeast transformants were selected on MD medium (1.3% YNB, 4 mg mL^−1^ biotin, 2% glucose). Expression of proteins in *P. pastoris* transformants was analyzed on BMM methanol-based medium (1 L flask, with the addition of 1 mL-methanol/day) after growing the yeast cells in BMG, as described previously [4]. Yeast growth was monitored spectrophotometrically at a wavelength of 600 nm (OD_600_), and protein concentration was determined using NanoDrop ND-1000 (Thermo Scientific (Wilmington, DE, USA) at 280 nm. At the maximum time of protein induction (6 days), cells were removed by centrifugation (6000× *g* for 15 min), and extracellular fraction was concentrated (if required) using 10-kDa MWCO PES membranes and a Vivaflow 50 system (Sartorius, Gottingen, Germany). The *Escherichia coli* DH5α strain was used as host for DNA manipulations using the standard techniques.

In this work, plasmid CHIT33-pIB4, a derivative of pIB4 (His4) including the methanol-regulated alcohol oxidase promoter (*AOX*1p) and gene *chit33* (X80006.1), which was obtained in a previous work [4], was used for creation of different variants of Chit33 using site-directed mutagenesis techniques. Phusion high-fidelity DNA polymerase (NEB, Ipswich, UK) and a pair of specific primers (Table S5 in Supplementary Material) including mutations responsible for substitutions S37D, D117W, S118Y, Q199S, N225S, N226R and R274S were used. The PCR conditions were: (i) 98 °C for 30 s; (ii) 25 cycles of 98 °C for 10 s, 55 °C for 30 s and 72 °C for 140 s; and (iii) final extension at 72 °C for 600 s. The PCR reactions were treated with *Dpn*I to eliminate the parental plasmid and transformed into *E. coli*. Mutations and integrity of the constructions were verified by DNA sequencing using the following primers: AOX1: 5′-GACTGGTTCCAATTGACAAGC-3′ and AOX2: 5′-CCTACAGTCTTACGGTAAACG-3′, both from Sigma Aldrich (St. Louis, MO, USA). Constructions including the desired mutation were linearized with *Stu*I (into *His4* of the pIB4 derivatives), transformed in *P. pastoris* by electroporation and confirmed by PCR as described in [4].

### 3.3. Hydrolytic Activity

The chitinase activity of the Chit33 variants was determined by detection of reducing sugars from chitinolytic materials using the 3,5-dinitrosalicylic acid (DNS) assay as explained previously [4]. Briefly, the reactions were performed in 1.5 mL Eppendorf tubes by adding 100 µL of enzymatic solution (undiluted) to 400 µL of 1% (*w*/*v*) colloidal chitin and other substrates (all at pH 5.0). Tubes were incubated at 900 rpm in a Thermo Shaker TS-100 (Boeco, Hamburg, Germany) for 1 h at 45 °C. The reactions were stopped at 100 °C for 10 min by adding 0.2 M NaOH to inactivate the enzymatic activity and to precipitate the remaining non-hydrolyzed material by using polysaccharides as a substrate. A calibration curve of NAG (0–3 mg mL^−1^) was used. One unit of activity (U) corresponded to the release of one µmol of reducing sugars per minute. Kinetic constants were determined using 0.1–15 mg mL^−1^ of colloidal chitin as a substrate. For NAG6, 25–1500 µM was used. Plotting and analysis of the curves was carried out using GraphPad Prism software (version 8.0), and the kinetic parameters were calculated by fitting the initial rate values to the Michaelis–Menten equation. Standard errors were obtained by fitting the normalized equation as v = (*k*_*cat*_/*K*_*m*_)[S]/(1 + [S]/*K*_*m*_).

### 3.4. Transglycosylation Activity and Mass Spectrometry Analysis

Transglycosylase reactions were carried out using NAG6 as substrate with 0.032 mg mL^−1^ of the corresponding chitinase variant and 2.5 mg mL^−1^ substrate in 100 mM phosphate potassium at pH 5.5 (total volume, 0.5 mL). Reactions were incubated at 900 rpm in a thermo shaker for 24 h at 45 °C as described above and then stopped by adding 0.2 M NaOH.

The molecular weight of produced the COS was assessed by MALDI-TOF-MS using a mass spectrometer with Ultraflex III TOF/TOF (Bruker, Billerica, MA, USA) and an NdYAG laser. Registers were taken in positive reflector mode within a mass interval of 40−5000 Da, external calibration and 20 mg mL^−1^ 2,5-dihydroxybenzoic in acetonitrile (3:7) as a matrix. Samples were mixed with the matrix in a 4:1 ratio, and 0.5 μL was analyzed. The amounts of NAG oligomers were deduced based on the MS peak areas generated by each oligomer, and the percentage of their relative abundance referred to the total peak areas detected for all NAG oligomers detected in the reaction.

### 3.5. Crystallization and X-ray Structural Determination

Initial crystallization conditions for wild-type Chit33 (20 mM Tris-HCl pH 7.0 and 50 mM NaCl) were explored with high-throughput techniques with a NanoDrop robot (Innovadyne Technologies Inc., Wilmington, DE, USA) using the sitting-drop vapor-diffusion method in MRC 96-well crystallization plates (Molecular Dimensions, Sheffield, UK). Five different commercial screens were tested (PACT and JCSG + Suites from Qiagen (QIAGEN N.V., Venlo, The Netherlands): JBScreen Classic 1–4 from Jena Bioscience (Jena, Germany), as well as Index and SaltRx packages from Hampton Research (Aliso Viejo, CA, USA)), using 9 to 29 mg mL^−1^ protein concentration and 1:1 or 2:1 (protein: reservoir) rates. The first crystals grew after four months in the commercial screen JCSG + Suites, from 24% PEG 1500, 20% glycerol. A streak-seeding technique accelerated the crystallization process to approximately two weeks, yielding the best diamond-shape crystals with 20% PEG 1500, 20% glycerol and 29 mg mL^−1^ protein concentration at a 1:1 ratio. To obtain the Chit33-D165A/E167A-NAG4 complex, the double mutant (24.9 mg mL^−1^, 2:1) was crystallized in 30% PEG 1500, 20% glycerol with the microseeding technique. Then, the best-quality crystal was transferred to a mother liquor solution supplemented with 18 mM of commercial tetra-acetyl chitotetraose (NAG4), Megazyme. After 30 min, the crystal was captured in liquid nitrogen for cryocooling.

Diffraction data were collected at the ALBA synchrotron station (Barcelona, Spain). The X-ray images were processed with XDS [26] and merged using Aimless from the CCP4 [27] suite package. Both structures were indexed in the P 2_1_ 2_1_ 2_1_ space group, with only one molecule in the asymmetric unit (51% of solvent content) at 1.12 Å (wild type) and 1.60 Å (NAG4 complex) resolution. The wild-type Chit33 structure was solved by molecular replacement using the atomic coordinates of its homologue, 2UY2 (45% sequence homology), with the MOLREP program in the CCP4 package [28]. Crystallographic refinement of both crystals was performed using the program REFMAC within the CCP4 suite [29]. Moreover, the model was further completed with the program Coot [30], combined with additional rounds of refinement in REFMAC. The figures were generated with PyMOL (PyMOL Molecular Graphics System, Version 2.0, Schrödlinger, LLC) [31]. Full crystallographic details are presented in Table S1 in Supplementary Material.

### 3.6. Molecular Dynamics

The co-ordinates of the double-mutant Chit33-D165A/E167A were reverted to native by changing the corresponding alanine residues to aspartate and glutamate, and Ser118 was mutated to tyrosine. The NAG6 ligand was designed using the NAG4 molecule captured in the Chit33-D165A/E167A crystal, and NAG moieties at positions −3 and −4 extrapolated by superposition to the reported hevamine complex (PDB: 1KR1) as a template. Molecular dynamic calculations were performed using the simple molecular dynamics module within Phenix [32] to shake up the complex Chit33- S118Y-NAG6 co-ordinates. A very short molecular dynamic at 300 K was performed.

## 4. Conclusions

Our structure–function analysis broadens our knowledge of the molecular mechanisms underlying the hydrolysis of chitinous material by plant-type fungal chitinases and offers a basis for development of the biotechnological applications of Chit33 and other GH18 *endo*-chitinases. In summary, the major structural trait of the Chit33 binding site is the existence of Asp117 and Arg274, which clamp the bended conformation of the substrate that occupies the full catalytic groove, conversely to that reported in the homologue hevamine. Moreover, Asn39 provides additional putative binding subsites at the aglycone, suggesting that NAG4 is the major product obtained from chitin hydrolysis. To analyze the role of the individual residues involved in substrate binding and catalysis, single-point mutations were performed in the Chit33 sequence, and the activities and kinetic parameters of the mutants were measured and compared to those of the wild-type enzyme. Kinetic assays show that the affinity for NAG6 is significantly compromised in all mutants, which is consistent with their involvement in making the polar links described in the crystallographic complex. Remarkably, the mutant S118Y exhibits an evidently superior hydrolytic performance against all the tested substrates, as well as a detectable transglycosylating capacity. Based on molecular dynamics simulation, a role as a switch facilitating the substrate entrance/releasing events was assigned to this Tyr, which may provide a rationale for the superior hydrolytic activity of this mutant as compared to the wild type. Additionally, increased retention of the glycosyl fragment at the active site of the −3 mutant might favour transglycosylation due to a stabilized intermediate state. Therefore, the S118Y mutant provides a promising platform for generation of novel engineered Chit33 variants with tailored function on chitinous material, as well as designed substrate specificity to produce novel COSs for various applications.

## Figures and Tables

**Table 1:** Kinetic parameters on colloidal chitin (A) and NAG6 (B) of the referred variants.

A	Colloidal Chitin			
Variant	*K_m_* (mg mL^−1^)	V_max_ (μmol min^−1^)	*k_cat_* (s^−1^)	*k_cat_*/*K_m_* ( s^−1^ mg^−1^ mL)
Wt	1.2 ± 0.2	0.05 ± 0.004	2.2 ± 0.169	1.8 ± 0.6
S37D	6.5 ± 1.1	0.2 ± 0.007	5.3 ± 0.167	0.8 ± 0.1
D117W	0.8 ± 0.1	0.15 ± 0.004	0.8 ± 0.02	0.9 ± 0.1
S118Y	0.6 ± 0.01	0.003 ± 0.0002	3.3 ± 0.22	5.5 ± 0.4
Q199S	0.1 ± 0.01	0.14 ± 0.02	0.1 ± 0.013	0.9 ± 0.1
N225S	9.1 ± 1.1	0.37 ± 0.06	15.0 ± 2.4	1.6 ± 0.3
N226R	0.6 ± 0.07	0.17 ± 0.003	0.3 ± 0.005	0.5 ± 0.06
R274S	6.0 ± 1.3	0.6 ± 0.06	10 ± 1.0	1.7 ± 0.4
**B**	**NAG6**			
**Variant**	***K_m_*** **(μM)**	**V_max_ (μmol min^−1^)**	***k_cat_*** **(s^−1^)**	***k_cat_*** **/*K_m_* (s^−1^ μM^−1^)**
Wt	346 ± 94	0.0017 ± 0.0002	189 ± 22	0.55 ± 0.06
S37D	1464 ± 124	0.0086 ± 0.0005	959 ± 56	0.65 ± 0.04
D117W	1360 ± 133	0.0066 ± 0.0004	736 ± 45	0.54 ± 0.03
S118Y	1439 ± 227	0.0129 ± 0.0015	1438 ± 167	1.00 ± 0.1
Q199S	995 ± 225	0.0092 ± 0.0013	1026 ± 145	1.03 ± 0.1
N225S	1021 ± 274	0.0092 ± 0.0016	1026 ± 178	1.00 ± 0.17
N226R	1678 ± 574	0.0096 ± 0.0023	1070 ± 256	0.64 ± 0.1
R274S	774 ± 399	0.0085 ± 0.0028	949 ± 312	1.23 ± 0.4

**Table 2:** Relative abundance of COS (%) in the referred Chit33 variants after 4 h reactions.

COS	Wt	S37D	D117W	S118Y	Q199S	N225S	N226R	R274S
**NAG2**	35.5	15.1	11.0	0	19.9	5.8	7.5	5.4
**NAG3**	13.9	19.6	7.4	0.3	8.8	2.2	5.0	1.2
**NAG4**	49.0	62.1	79.1	0.6	64.2	76.2	83.2	89.3
**NAG5**	1.0	1.9	1.2	1.5	1.6	4.0	2.3	1.8
**NAG6**	0.6	1.3	1.3	97.6	10.5	11.8	2.0	2.3

## Data Availability

The co-ordinates and structural factors of Chit33 and its complex with NAG4 were deposited in the Protein Data Bank with the accession codes 7ZYA and 7ZY9, respectively.

## Supplementary Materials

The following supporting information can be downloaded at: https://www.mdpi.com/article/10.3390/ijms23147599/s1.

## Abbreviations

GH: glycosyl hydrolase; COS, chito-oligosaccharides: NAG, N-acetyl glucosamine; Chit33, endo-chitinase from *Trichoderma harzianum*.
