# Supplementary material for: Structure–Function Insights into the Fungal Endo-Chitinase Chit33 Depict its Mechanism on Chitinous Material
Source: Int J Mol Sci. 2022 Jul 9;23(14):7599. doi: 10.3390/ijms23147599 (PMC9323625; doi:10.3390/ijms23147599)
Supplement: Supplementary file 1 [file ijms-23-07599-s001.zip › ijms-1808142-supplementary.pdf]

Supplementary Materials

Structural and biochemical analysis of the fungal endo-quitinase Chit33 depicts its mechanism on chitinous material

Elena Jiménez-Ortega[a^]. Peter Elias Kidibule[b^]. María Fernández-Lobato[b*] and Julia Sanz-Aparicio[a*]

[a] Department of Crystallography and Structural Biology. Institute of Physical-Chemistry Rocasolano. CSIC. 28006 Madrid. Spain

[b] Department of Molecular Biology. Centre of Molecular Biology Severo Ochoa. CSIC-UAM. 28049 Madrid. Spain

**Table S1**	Crystallographic statistics
**Table S2**	Specific activity on the referred chitosan types
**Table S3**	Peak areas of the NAG series detected by MS in reactions mediated with the Chit33 variants and NAG6
**Table S4**	Peak areas of the NAG series detected by MS in reaction mediated by the Chit33 variants and NAG6 after 24h reactions
**Table S5**	Primers used for site-directed mutagenesis
**Figure S1**	Michaelis-Menten kinetics for the Chit33 variants on colloidal chitin and NAG6

TABLE S1. Crystallographic statistics (values in parentheses are for the high-resolution shell).

**Crystal data**	Chit33	Chit33 D165A/E167A - NAG4
Space group	$P\ 2_1\ 2_1\ 2_1$	$P\ 2_1\ 2_1\ 2_1$
Unit cell parameters		
a (Å)	65.61	65.42
b (Å)	66.91	66.78
c (Å)	75.32	76.44
**Data collection**		
Beamline	ALBA XALOC	ALBA XALOC
Temperature (K)	100	100
Wavelength (Å)	0.979190	0.979180
Resolution (Å)	46.85-1.12 (1.14-1.12)	46.73- 1.60 (1.63-1.60)
**Data processing**		
Total reflections	809980 (38863)	297035 (14842)
Unique reflections	127452 (6255)	44920 (2190)
Multiplicity	6.4 (6.2)	6.6 (6.8)
Completeness (%)	99.8 (99.9)	100.0 (100.0)
Mean $I/\sigma(I)$	20.9 (2.6)	12.8 (3.1)
$R_{merge}^{\dagger}$ (%)	3.7 (69.8)	8.3 (62.3)
$R_{pim}^{\dagger\dagger}$ (%)	1.6 (30.5)	3.5 (25.5)
Molecules per ASU	1	1
**Refinement**		
R_{work} / $R_{free}^{\dagger\dagger\dagger}$ (%)	14.97/16.04	14.47/16.84
**Nº of atoms/average B** ($Å^2$)		
Protein	2390/16.48	2350/17.82
Ligands	27/29.28	57/39.70
Water Molecules	371/32.45	302/34.40
All atoms	2788/18.73	2709/20.13
**Ramachandran plot** (%)		
Favoured	99	99
Outliers	0	0
**RMS deviations**		
Bonds (Å)	0.0088	0.0090
Angles (°)	1.4664	1.4984
**PDB accession codes**	7ZYA	7ZY9

$^{\dagger}R_{merge} = \sum_{hkl} \sum_i | I_i(hkl) – [I(hkl)] | / \sum_{hkl} \sum_i I_i(hkl)$. where $I_i(hkl)$ is the ith measurement of reflection hkl and [I(hkl)] is the weighted mean of all measurements.

$^{\dagger\dagger}R_{pim} = \sum_{hkl} [1/(N - 1)] 1/2 \sum_i | I_i(hkl) – [I(hkl)] | / \sum_{hkl} \sum_i I_i(hkl)$. where N is the redundancy for the hkl reflection.

$^{\dagger\dagger\dagger}R_{work} / R_{free} = \sum_{hkl} | Fo – Fc | / \sum_{hkl} | Fo |$. where Fc is the calculated and Fo is the observed structure factor amplitude of reflection hkl for the working / free (5%) set. respectively.

TABLE S2. Specific activity on the referred chitosan types.

Variant	Colloidal chitin (MW n.d.. DD ≤8)	CHIT50 (50-190 kDa. DD 77%)	CHIT100 (100-300 kDa. DD >90%)	CHIT600 (600-800 kDa; DD >90%)
**Wt**	6.7 ±0.2	19.5 ±0.3	2.6 ±0.03	n.a.
**S37D**	1.7 ±0.1	10.0 ±0.3	2.0 ±0.02	0.3±0.001
**D117W**	1.9 ±0.1	5.1 ±0.1	0.9 ±0.01	n.a.
**S118Y**	15.3 ±0.7	94.4 ±4.1	19.4 ±0.6	n.a.
**Q199S**	4.7 ±0.1	9.6 ±0.6	9.6 ±0.3	n.a.
**N225S**	4.6 ±0.2	21.5 ±1.0	3.4 ±0.04	n.a.
**N226R**	4.1 ±0.1	7.9 ±0.2	0.8 ±0.1	n.a.
**R274S**	2.2 ±0.1	13.8 ±0.3	1.8 ±0.1	n.a.

Specific activity (U mg^{-1}) using the referred chitosan as substrates. Reactions were performed in triplicate and standard errors are indicated; n.d.. no determined; n.a.. no activity detected; DD. Deacetylation Degree.

TABLE S3. Peak areas of the NAG series detected by MS in reaction mediated by the Chit33 variants and NAG6

Wt

m/z	Peak area				
	0h	1h	2h	3h	4h
447.169894	n.d.	522.872209	1359.00907	361.415596	703.847088
650.249034	n.d.	195.136836	782.992968	179.208595	274.885643
853.300374	7.06934849	1590.46216	5149.23702	880.612962	972.332333
1056.35708	26.6680583	18.0583894	46.026739	12.9098203	20.1980323
1259.38343	3273.49823	11.9109029	40.7508395	6.28328536	11.0673346
1462.41891	145.546507	16.7617562	44.5422867	14.8613305	11.4190856

S37D

		Peak area			
m/z	0h	1h	2h	3h	4h
447.192316	n.d.	362.734166	1250.811333	305.2678083	257.662283
650.281472	n.d.	409.926336	1272.962799	307.7697653	333.30445
853.345294	64,43226753	1840.40012	584.8224769	1035.864024	1056.01211
1056.40259	71.54345845	138.148386	49.32729074	24.75259189	31.7873075
1259.45843	6264.110586	37.6724238	54.30709408	21.09789333	22.8453215
1462.50303	355.053145	41.152849	13.49450185	20.06447915	24.0221646

D117W

	Peak area				
m/z	0h	1h	2h	3h	4h
447.20048	n.d.	464.531303	918.916208	254.160192	212.765565
650.287627	n.d.	117.105589	538.801529	161.95989	143.465423
853.362565	64.43226753	916.299241	1929.92123	1822.93609	1530.47401
1056.37912	7.,54345845	17.6936387	47.7296729	30.3694751	23.8170379
1259.47589	6264.110586	17.59809	31.0332072	27.8820061	12.8861343
1462.5402	355.053145	14.5567186	29.9265201	58.8780017	39.9026678

S188Y

	Peak area				
m/z	0h	1h	2h	3h	4h
447.20048	n.d.	334.4162376	770.41187	625.765907	262.33374
650.287627	n.d.	364.3165198	3239.00524	523.576211	23.0860787
853.362565	7.06934849	3753.398245	3955.31727	3073.93893	44.2168977
1056.37912	26.6680583	65.1840015	243.752879	33.0368787	103.037357
1259.47589	3273.49823	261.1205172	228.899595	86.0634975	6836.40056
1462.5402	145.546507	178.2737261	122.749984	39.6683242	295.949741

N225S

	Peak area			
m/z	0h	1h	3h	4h
447.170196	9.33917182	23.7897087	141.295411	343.420901
650.266862	7.50137177	18.9234566	51.3080831	133.338918
853.325272	19.6915993	116.448142	953.070876	4533.06521
1056.36587	24.8783619	37.5698863	61.7707671	236.734565
1259.42283	2667.76251	3872.86533	3502.52733	703.899828
1462.47677	143.608013	218.790456	187.649081	470.96124
1665.50949	5.61644704	12.4031181	18.6988078	32.3127751

N226R

	Peak area			
m/z	0h	1h	3h	4h
447.178451	9.33917182	660.155336	852.508175	483.689469
650.265609	7.50137177	330.104718	279.539936	318.400036
853.322462	19.6915993	7823.4396	4201.52711	5338.41851
1056.37306	24.8783619	167.400299	46.0913735	150.238644
1259.41735	2667.76251	247.68366	44.1802842	128.912331
1462.46197	143.608013	315.096422	50.8607334	174.197163
1665.50184	5.61644704	23.6120261	n.d.	15.1370528

R274S

	Peak area		
m/z	0h	2h	4h
447.170196	9.33917182	108.242097	255.397213
650.266862	7.50137177	31.1653827	56.5724428
853.325272	19.6915993	1989.85551	4256.61505
1056.36587	24.8783619	43.4389663	87.3775622
1259.42283	2667.76251	64.6398262	108.63619
1462.47677	143.608013	101.369156	153.498171
1665.50949	5.61644704	13.8739912	6.00257681

Only $[M+Na]^+$ peaks corresponding to the NAG series detected in the reactions with the referred variants are indicated; m/z of 447.170196, 650.265609, 853.325272, 1056.36587, 1259.42283 and 1462.47677 for NAG2, NAG3, NAG4, NAG5, NAG6, NAG7; n.d., no detected.

TABLE S4. Peak areas of the NAG series detected by MS in reaction mediated by the Chit33 variants and NAG6 after 24h reactions

**m/z**	**COS**	**Peak area**						
		**Wt**	**S37D**	**D117W**	**S118Y**	**Q199S**	**N225S**	**N226R**
447,189996	NAG2	1297.7684	1250.81133	918.916208	564.434407	852.508175	622.261403	3225.28022
650,272402	NAG3	1228.20004	1272.9628	538.801529	766.824092	279.539936	274.841567	2124.57075
853,343227	NAG4	242.312725	584.822477	1929.92123	3178.36173	4201.52711	2005.13701	4195.44749
1056,39781	NAG5	62.6923724	49.3272907	47.7296729	87.1408261	46.0913735	48.5593615	117.185437
1259,45199	NAG6	35.3337893	54.3070941	31.0332072	96.4479803	44.1802842	210.03166	83.7368256
1462,51017	NAG7	27.1913441	33.3051667	29.9265201	84.3497867	50.8607334	57.8035547	81.7964712

Only [M+Na] + peaks corresponding to the NAG series detected in the reactions with the referred variants are indicated

TABLE S5. Primers used for site-directed mutagenesis.

Oligonucleotide	Mer	Sequence
S37D(+)	30	GGTCAGAAC**GAT**GCCAACTCGCAAAGCACC
S37D(-)	30	CGAGTTGGC**ATC**GTTCTGACCCCAGTATAC
D117W(+)	30	TCTCGGCGGC**TGG**TCTTACACTCAGGGCGG
D117W(-)	30	GAGTGTAAGA**CCA**GCCGCCGAGAGAAAGGA
S118Y(+)	27	CGGCGGCGAT**TAC**TACACTCAGGGCGG
S118Y(-)	27	CTGAGTGTA**GTA**ATCGCCGCCGAGAGA
D165AE167A(+)	36	TTCGACTTC**GCT**TTT**GCT**GCTACCACCAACAACCTC
D165AE167A(-)	42	GGTAGC**AGC**AAA**AGC**GAAGTCGAAGCCATCGACGACGGCACT
Q199S(+)	31	CTGCTGCTCCC**TCT**TGCTTCTTCCCCGACGC
Q199S(-)	30	GGAAGAAGCA**AGA**GGGAGCAGCAGAGAAGT
N225S(+)	32	TCAGTTCTAC**TCT**AACCCCTGTGGCGTCAGCG
N225S(-)	32	CACAGGGGTT**AGA**GTAGAACTGAATCTGGATC
N226R(+)	33	CAGTTCTACAAC**TCT**CCCTGTGGCGTCAGCGGC
N226R(-)	36	CTGACGCC**ACA**GGGAGAGTTGTAGAACTGAATCTGG
R274S(+)	26	AGGTGCTGGT**TCT**GGCTACGTCTCTG
R274S(-)	29	GACGTAGCC**AGA**ACCAGCACCTGGGCCAG

Substitutions made are in bold

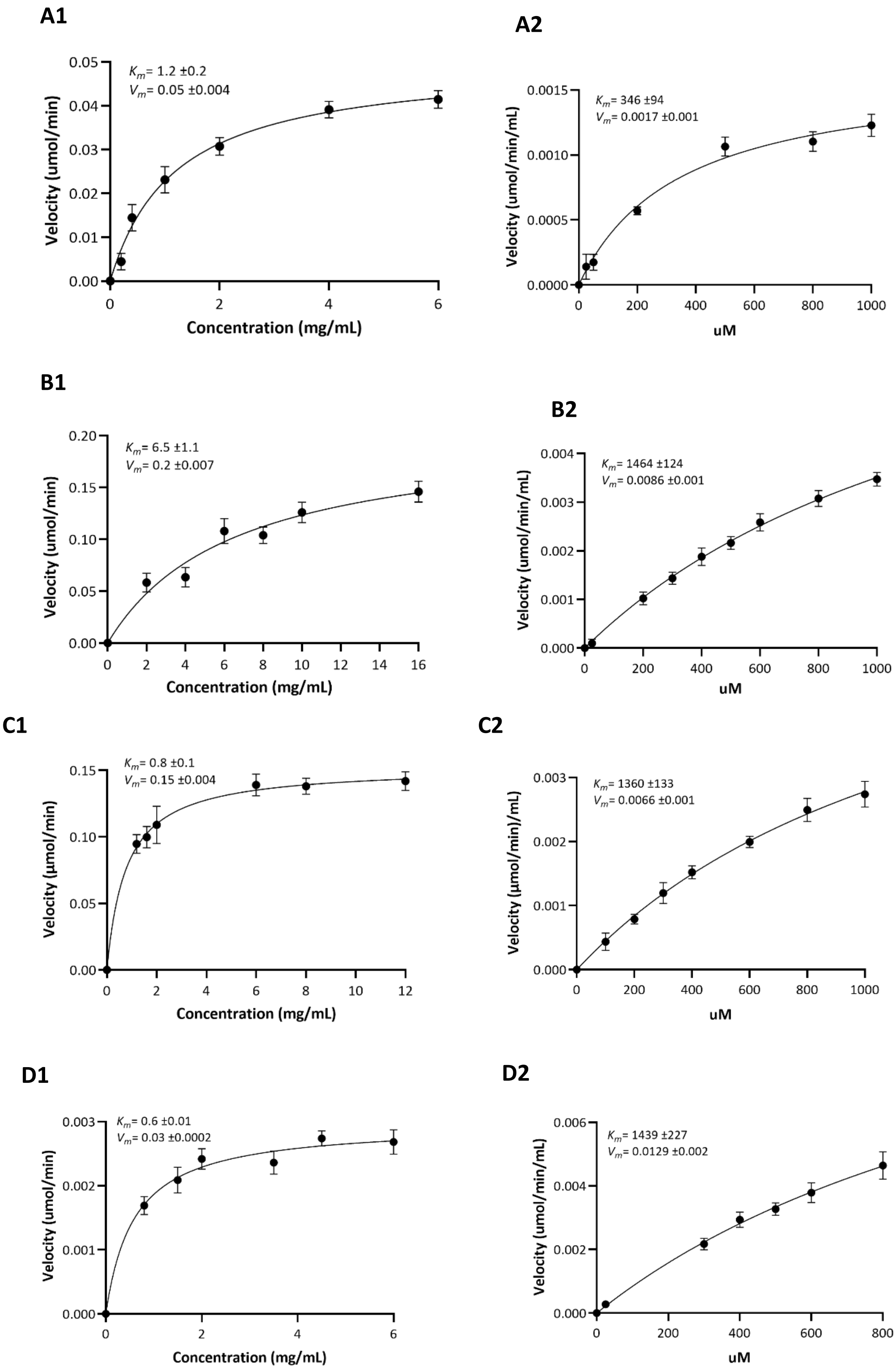

A1
Km= 1.2 ±0.2
Vm= 0.05 ±0.004
Velocity (umol/min)
Concentration (mg/mL)
A2
Km= 346 ±94
Vm= 0.0017 ±0.001
Velocity (umol/min/mL)
uM
B1
Km= 6.5 ±1.1
Vm= 0.2 ±0.007
Velocity (umol/min)
Concentration (mg/mL)
B2
Km= 1464 ±124
Vm= 0.0086 ±0.001
Velocity (umol/min/mL)
uM
C1
Km= 0.8 ±0.1
Vm= 0.15 ±0.004
Velocity (µmol/min)
Concentration (mg/mL)
C2
Km= 1360 ±133
Vm= 0.0066 ±0.001
Velocity (µmol/min)/mL)
uM
D1
Km= 0.6 ±0.01
Vm= 0.03 ±0.0002
Velocity (umol/min)
Concentration (mg/mL)
D2
Km= 1439 ±227
Vm= 0.0129 ±0.002
Velocity (umol/min/mL)
uM

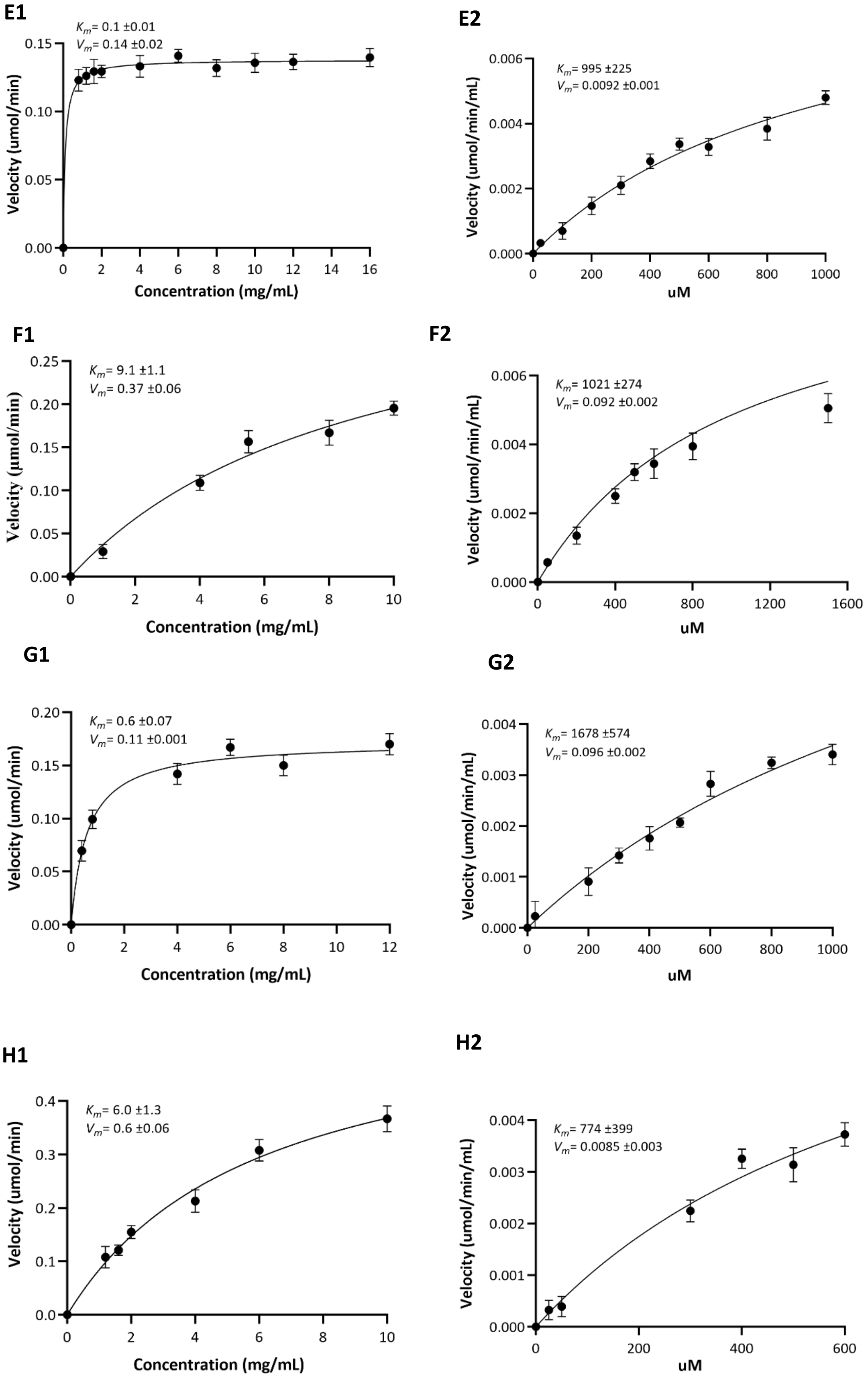

Figure S1. Michaelis-Menten kinetics for the Chit33 variants on colloidal chitin and NAG6. Colloidal chitin (1, left) and NAG6 (2, right). A, wt; B, S37D; C, D117W; D, S118Y; E, Q199S; F, N225S; G, N226R; H, R274S. Vmax and *K*m values (from GraphPad Prism software version 8.0) are also shown. All reactions were performed in triplicate and the kinetic parameters were calculated fitting

the initial rate values to the Michaelis-Menten equation. Standard errors were obtained by fitting the normalized equation as $v = (k_{cat}/K_m) [S]/(1 + [S]/K_m)$.
